# Sero-epidemiological survey and risk factors associated with brucellosis in dogs in south-western Nigeria

**DOI:** 10.11604/pamj.2016.23.29.7794

**Published:** 2016-02-04

**Authors:** Modupe Comfort Ayoola, Akwoba Joseph Ogugua, Victor Oluwatoyin Akinseye, Tunde Olu Joshua, Morenikeji Folusho Banuso, Folashade Julianah Adedoyin, Hezekiah Kehinde Adesokan, Temidayo Olutayo Omobowale, John Olusoji Abiola, Patricia Ihuaku Otuh, Helen Oyebukola Nottidge, Emma-Jane Dale, Lorraine Perrett, Andrew Taylor, Judy Stack, Simeon Idowu Babalola Cadmus

**Affiliations:** 1 Department of Veterinary Public Health and Preventive Medicine, Faculty of Veterinary Medicine, University of Ibadan, Ibadan, Nigeria; 2 Department of Veterinary Medicine and Surgery, College of Veterinary Medicine, Federal University of Agriculture, Abeokuta, Nigeria; 3 Department of Veterinary Medicine, Faculty of Veterinary Medicine, University of Ibadan, Ibadan, Nigeria; 4 Department of Bacteriology, Animal & Plant Health Agency, United Kingdom

**Keywords:** Brucellosis, dogs, sero-epidemiology, risk factors, Nigeria

## Abstract

**Introduction:**

In Nigeria, there is limited information on brucellosis particularly in dogs, despite its public health implications. We undertook a sero-epidemiological survey of brucellosis in dogs to determine the prevalence of the disease and associated risk factors for its occurrence in Nigeria.

**Methods:**

A cross-sectional study was conducted to screen dogs in south-western Nigeria for antibodies to *Brucella sp* using the rapid slide agglutination test (RSA) and Rose Bengal test (RBT), with positive samples confirmed respectively by serum agglutination test (SAT) and competitive enzyme linked immunosorbent assay (cELISA). Data were analyzed with STATA-12.

**Results:**

From the 739 dog sera tested, 81 (10.96%) were positive by RSA and 94 (12.72%) by RBT; these were corroborated with SAT (4/81; 4.94%) and cELISA (1/94; 1.06%), respectively. Logistic regression identified location (OR=0.04; 95% CI: 0.02-0.09), breed (OR=1.71; 95% CI: 1.34-2.19), age (OR=0.10; 95% CI: 0.04-0.30) and management system (OR=8.51; 95% CI: 1.07-68.05) as risk factors for Brucella infection by RSA. However, location (OR=10.83; 95% CI: 5.48-21.39) and history of infertility (OR=2.62; 95% CI: 1.41-4.84) were identified as risk factors using RBT.

**Conclusion:**

Given the 10.96% to 12.72% seroprevalence of brucellosis recorded in this study, we advocate control of the disease in dogs, and public health education for those at risk of infection. Again, further studies are required to elucidate the role of dogs in the epidemiology of brucellosis in Nigeria considering the conducive human-animal interface and ecological factors responsible for the transmission of the disease.

## Introduction

Brucellosis is an infectious disease of global public health importance, with far reaching economic impact since it is associated with reproductive losses in animals [1]. The aetiological agent of the disease is the bacteria of the genus *Brucella*. Generally in canines, *Brucella canis* is the main aetiological agent. It has a ubiquitous distribution and has been reported in the United States, Canada, Central and South America, Tunisia, South Africa, Nigeria, Madagascar, Malaysia, India, Korea, Japan and China among others [2–7]. The organism is not found in New Zealand and Australia [8]. However, brucellosis in dogs can also be caused by *B. abortus, B. suis and B. melitensis* [9–12] where dogs are in close contact with cattle, sheep, goats and pigs and inadvertently share the same environment. Transmission of *Brucella* infection in dogs occurs via ingestion of contaminated materials or venereal routes [12]. It can also be easily transmitted among dogs reared intensively in breeding kennels or where owners rear two or more dogs. In addition, dogs fed on foetal wastes and raw meats from abattoirs have been reported to be infected with *B. abortus* [13]. The clinical manifestation of the disease in dogs includes abortion, infertility, orchitis, epididymitis and testicular atrophy, among others [14, 15]. Laboratory diagnosis of the disease can be achieved by various serological tests; including the rapid slide agglutination (RSA), indirect fluorescent antibody, serum agglutination test (SAT), agar gel immuno-diffusion assay (AGID) and enzyme linked immunosorbent assay (ELISA). Other tests used are Rose Bengal plate test (RBT), complement fixation tests and fluorescent polarisation assay. However, false positive results may occur due to cross reacting antibodies from other Gram-negative organisms with RBT and RSA [5, 16–18].

While several studies have been conducted on the epidemiology of brucellosis, especially in cattle in Nigeria [19–21], only little is known of the disease among dogs in the country. Whereas, the practice of feeding dogs with foetuses and raw meat from slaughtered cattle coupled with influx of unregistered and suspected brucellosis infected dogs from foreign countries is common. Worse still, there is increasing ownership of dogs by people with poor knowledge of brucellosis, complicated by poor and deplorable health situations in most dog kennels in Nigeria. Based on the aforementioned, it becomes imperative to carry out an epidemiological survey of brucellosis in dogs in Nigeria towards providing empirical data for its control in dogs and humans. To achieve this, we set out to determine the seroprevalence and risk factors associated with brucellosis in dogs in south-western Nigeria.

## Methods

### Study setting

**Figure 1:**
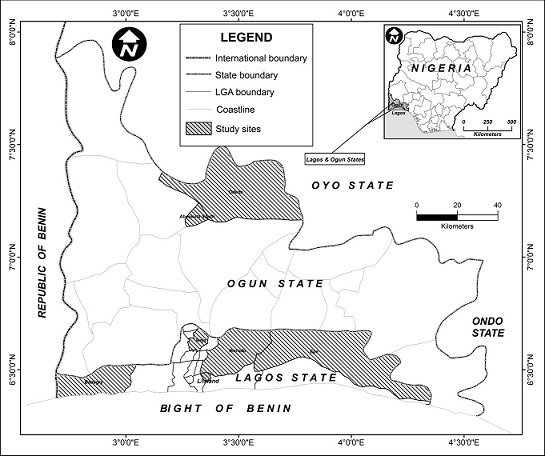
Study areas: Lagos and Ogun States (Inset: Nigeria)

The study was conducted in Lagos and Ogun States, south-western Nigeria. Lagos State (Figure 1) is an administrative division of Nigeria, located in the south-western part of the country and the smallest in land area of Nigeria′s 36 states [22]. It is arguably the most economically important state of the country, containing Lagos Division, the nation′s largest urban area. Ogun State is another state in south-western Nigeria, located in the north and slightly east to Lagos. Given its contiguous location to Lagos and neighbouring African countries, it also plays vital economic and trans-border activities relating to animal movements and by implication, trans-border diseases. Dogs are reared in both states as pets, and for security as well as for economic purposes.

### Study design

We carried out a cross-sectional study. Data from sero-epidemiological survey of dogs presented to major veterinary hospitals/clinics in Lagos and Ogun States were collected between July 2011 and February 2014 for antibodies to *Brucella sp*. In addition, epidemiological data from hunting and stray dogs screened in Ogun State were obtained.

### Sample collection and storage

About 5 ml of blood was aseptically collected through the cephalic vein of each sampled dog by a veterinarian. The breed, sex and age of the dogs as well as feed type, and reproduction-related history were obtained and recorded accordingly. The samples were transported to the Tuberculosis and Brucellosis Laboratories of the Department of Veterinary Public Health and Preventive Medicine, University of Ibadan, Nigeria in a cooler. The blood samples were allowed to clot and centrifuged at 3000 x g for five minutes. Serum samples were decanted and stored at -20^o^C until they were assayed.

### Test reagents and procedures

All test reagents used in this study (RSA antigen, RBT antigen, SAT antigen, cELISA kit) were supplied by the Animal and Plant Health Agency (APHA) (Surrey, United Kingdom) and standardized according to the stipulations set by the OIE [23]. Serum samples were examined by RSA, RBT, SAT and cELISA for antibodies to *Brucella sp*. The RSA test and RBT were performed as described by Amin et al. (2012) [24]. Briefly, 30µl of serum sample was mixed with equal volume of antigen on a white enamel plate. The plate was rocked and serum samples that showed agglutination were recorded as sero-positive to *Brucella sp*. Positive samples by RSA and RBT were further respectively analysed using SAT and cELISA as previously described [24, 25]. Data were analyzed using Stata versions 12. Frequencies were generated and Chi square test was used to explore variables potentially associated with *Brucella* infection among dogs. The level of statistical significance was set at p < 5%. Variables significant at 10% on bivariate analysis were entered into the logistic regression model.

## Results

Out of 739 dogs screened (Lagos State=385; Ogun State=354), an overall seroprevalence of 10.96% and 12.72% were recorded by RSA and RBT out of which, 4.9% (4/81) and 1.1% (1/94) were further supported by SAT and cELISA, respectively. About one third (34.1%) of the dogs screened were Alsatians, more than half (57.6%) were females while two third (65.8%) were adults.

### Rapid slide agglutination test

Of 739 serum samples examined by RSA, higher seroprevalence (20.1%) was obtained from Ogun State compared to those from Lagos State (2.6%) (Table 1). Breed-specific prevalence was highest among mongrels (38.9%), followed by the Boerboels (8.1%); while the least being the Rottweiler and Alsatian breeds of dogs (1.2%). Also, females had higher seropositivity (11.5%) to antibodies to *Brucella sp*. than the males (10.2%). Age-specific seroprevalence showed that dogs >3 years had higher seroprevalence (13.7%) than those <3 years (5.6%) (Table 1). More than one-tenth (12.7%) of dogs with history of infertility were seropositive to antibodies to *Brucella sp*. with only 4.5% from those without infertility. However, a higher seroprevalence of 11.5% was obtained among dogs without history of abortion with only 2.2% from those with previous abortion. Similarly, dogs fed with cow foetus/raw meat had lower seroprevalence (2.8%) than those unexposed to this feed type (13.5%). Seropositivity to antibodies to *Brucella sp* was also higher among confined (11.2%) than stray (4.3%) dogs (Table 1). The adjusted multivariate logistic regression identified factors like location (OR=0.1; 95% CI: 0.03 - 0.13) and age (OR=6.3; 95% CI: 3.3 - 11.6) to be associated with seropositivity to antibodies to *Brucella sp* (Table 2).

**Table 1:** Factors associated with sero-prevalence of brucellosis among dogs in Ogun and Lagos States, south-western Nigeria by RSA

Variable	Category	RSA		OR	95% CI	P-value
		Positive n (%)	Negative n (%)			
**Location**	Ogun State	71(20.1)	283 (79.9)	9.4	4.8 – 18.6	0.00
	Lagos State	10(2.6)	375 (97.4)	1		
**Breed**	Mongrel	65 (38.9)	102 (61.1)	1		
	Alsatian	3 (1.2)	245 (98.8)	0.01	0.006 – 0.10	0.01
	Boerboel	10 (8.1)	114 (91.9)	0.13	0.06 – 0.30	0.00
	Rottweiler	1 (1.2)	82 (98.8)	0.02	0.002 – 0.14	0.00
	Other	2 (1.7)	115 (98.3)	0.02	0.007 – 0.11	0.00
**Sex**	Male	32 (10.2)	281 (89.8)	1		
	Female	49 (11.5)	377 (88.5)	1.1	0.7 – 1.8	0.67
**Age**	<3years	14 (5.6)	237 (94.4)	1		
	>3years	67 (13.7)	421 (96.3)	2.7	1.5 – 4.9	0.00
**Mating**	Mated	50 (10.8)	413 (89.2)	1		
	Not mated	31 (11.2)	245 (88.8)	1.1	0.6 – 1.7	0.95
**Infertility**	Fertile	7 (4.5)	148 (95.5)	1		
	Infertile	74 (12.7)	510 (87.3)	3.1	1.3 – 6.8	0.01
**Abortion**	No	1 (2.2)	45 (97.8)	1		
	Yes	80 (11.5)	613 (88.5)	5.9	0.8 – 43.2	0.08
**Fed with fetus/ raw meat**	No	5 (2.8)	171 (97.3)			
	Yes	76 (13.5)	487 (86.5)	5.3	2.1 – 13.4	0.00
**Management system**	Confined	1 (4.3)	22 (95.7)			
	Stray	80 (11.2)	636 (88.8)	2.8	0.4 – 20.8	0.50

**Table 2:** Results of logistic regression analysis of factors associated with seroprevalence of brucellosis (with RSA) among dogs

Variable	Odds ratio	95% CI	p-value
**Location**			
Ogun State	1		
Lagos State	0.1	0.03 – 0.13	0.00
**Age**			
<3years	1		
>3years	6.3	3.3 – 11.6	0.00

### Rose Bengal test

From the 739 serum samples examined by RBT, a higher seroprevalence (21.8%) was recorded in Lagos than Ogun (2.8%). The Boerboel breed had the highest breed specific prevalence (21.8%) while the males (13.4%) were more seropositive to antibodies to *Brucella sp* than the females (12.2%) (Table 3). Seropositivity was also higher among dogs >3 years (16.2%) than those <3 years (6.0%); while, dogs without history of mating showed higher (14.5%) seroprevalence than those with such history (11.7%). Unexpectedly, lower seroprevalence were obtained among dogs with history of infertility (13.7%) and abortion 8.7%). Similarly, dogs fed with cow foetuses/raw meat (10.2%) and those confined (13.0%) showed higher seropositivity to antibodies to *Brucella sp* (Table 4). Overall, location (OR=11.2; 95% CI: 5.7 - 22.1) and infertility (OR=2.6; 95% CI: 1.4 - 4.8) were identified as factors associated with seropositivity to antibodies to *Brucella sp* using the RBT.

**Table 3:** Factors associated with sero-prevalence of brucellosis among dogs in Ogun and Lagos States, south-western Nigeria by RBT

Variable	Category	RBT		OR	95% CI	P-value
		Positive n (%)	Negative n (%)			
**Location**	Ogun State	10 (2.8)	344 (97.2)	1		
	Lagos State	84 (22.1)	301 (77.9)	9.6	4.8 – 18.8	0.00
**Breed**	Mongrel	8 (5.0)	159 (95.0)	1		
	Alsatian	34 (13.7)	214 (86.3)	3.2	1.4 – 7.0	0.01
	Boerboel	27 (21.8)	97 (78.2)	5.5	2.4 – 12.7	0.00
	Rottweiler	8 (9.6)	75 (90.4)	2.1	0.8 – 5.9	0.23
	Other	17 (14.5)	100 (85.5)	3.4	1.4 – 8.1	0.01
**Sex**	Male	42 (13.4)	271 (86.6)	1		
	Female	52 (12.2)	374 (87.8)	0.9	0.5 – 1.4	0.71
**Age**	<3years	15 (6.0)	236 (94.0)	1		
	>3years	79 (16.2)	409 (83.8)	3.1	1.7 – 5.4	0.00
**Mating**	Mated	54 (11.7)	409 (88.3)	1		
	Not mated	40 (14.5)	236 (85.5)	1.2	0.8 – 2.0	0.32
**Infertility**	Fertile	14 (9.0)	141 (91.0)	1		
	Infertile	80 (13.7)	504 (86.3)	1.6	0.9 – 2.9	0.16
**Abortion**	Yes	4 (8.7)	42 (91.3)	1		
	No	90 (13.0)	603 (97.0)	1.6	0.5 – 4.5	0.53
**Fed with fetus/ raw meat**	Yes	18 (10.2)	158 (89.8)	1		
	No	76 (13.5)	487 (86.5)	1.4	0.8 – 2.4	0.32
**Management system**	Stray	1 (4.3)	22 (95.7)	1		
	Confined	93 (13.0)	623 (97.0)	3.3	0.4 – 24.7	0.36

**Table 4:** Results of logistic regression analysis of factors associated with seroprevalence of brucellosis (with RBT) among dogs

Variable	Odds ratio	95% CI	p-value
**Location**			
**Ogun State**	1		
**Lagos State**	11.2	5.7 – 22.1	0.00
**Infertility**			
**Fertile**			
**Infertile**	2.6	1.4 – 4.8	0.01

## Discussion

We report for the first time a large population based brucellosis survey covering diverse dog populations under different settings in Nigeria. The importance of this study is connected with the backdrop of increased dog ownership and very little knowledge about risk factors associated with brucellosis in dogs and its related public health implications in Nigeria. In this study, we employed the use of two *Brucella* antigens namely the standardised RBT antigen and RSA test antigen. The RBT is known to be sensitive to *B. abortus* antibodies, while on the other hand, the RSA is sensitive to *B. canis*. The findings of 10.96% and 12.72% seroprevalence of brucellosis among dogs by RSA and RBT, respectively underscore the importance of this survey in the study area. Importantly, these findings reiterate previous reports [2, 26–28] that brucellosis is endemic in Nigeria. The seroprevalence obtained in this study may be attributed to the fact that brucellosis control programme is non-existent in Nigeria and vaccination of cattle (from locations where dogs could pick up *B. abortus* infection) against brucellosis is not practised [29, 30]. Another source of infection may be infected breeding dogs that shed *Brucella* organism and contaminate dog kennels [31]. Again, the seroprevalence of 10.96% recorded by RSA may not be unconnected with uncontrolled importation of infected dogs from countries with history of *B. canis* in their kennels. These findings portend significant public health implications following the practice of unregulated dog mating without prior screening for brucellosis (a common practice among dog breeders in Nigeria) and associated close contacts between dogs and humans. In addition, poor knowledge of brucellosis [32] coupled with unhygienic practices among dog owners are issues of public health concern that can enhance human infection.

The higher seropositivity recorded by RBT compared with RSA in this study, may be linked with the practice of feeding dogs with cow foetuses/raw meat which is common in the study setting [2]. Again, due to unhygienic practice of disposal of aborted foetuses by herdsmen found among West African countries [33], hunting or stray dogs in such environments may consume loads of *Brucella* organisms along with foetal wastes. Thus, this may lead to infection with *B. abortus* which is not a natural pathogen of dogs [10, 26]. Again, we found that seropositivity to *Brucella* infection among dogs sampled was associated with location of sampling, a finding similar to previous report [2]. As observed, dogs in Lagos were more than nine times more likely to be infected with *Brucella* organism than those in Ogun (using the RBT). This infers that majority of dogs screened in Lagos might be infected with *B. abortus*. This observation may be due to the common practice of importing exotic dogs which are not often screened at the point of entry from neighbouring countries to Lagos. Again since Lagos has a major international airport, the importation corridor therefore makes it easier for more unscreened imported dogs to enter Lagos and thus, the higher population of dog breeders in the state. The implication of this is that Lagos also has more dog breeders most of whom do not keep standard kennel practice; leading to the importation of infected dogs and feeding of dogs with abattoir meat waste (including those originating from cattle with *B. abortus* infection). These assertions are buttressed by Okoh et al. (1978) [34] who isolated *B. canis* from an imported breed of boxer dog in Kano, northern Nigeria; coupled with earlier findings of Cadmus et al. (2011) [2] that associated brucellosis in dogs to feeding of abattoir waste in dogs screened. Therefore, the higher seroprevalence of brucellosis in dogs from Lagos could be due to the more common practice of feeding dogs with foetal waste or raw meat in Lagos than Ogun [2].

Furthermore, our findings identified history of infertility as a factor associated with seropositivity of *Brucella* infection among dogs screened using RBT. Infertility and abortion have been previously reported as major risk factors in the epidemiology of brucellosis among dogs in Ahaz, Iran [6]. Again, infertility is one of the common signs of brucellosis in dogs as well as abortion, failure to conceive, still birth and birth of weak puppies [6, 35]. However, due to the fact that RBT antigen can indiscriminately detect antibodies from other cross reacting organisms, the infertility and abortion identified, to be associated with seropositivity by RBT among dogs screened, may have been due to other organisms. Again, age was identified as a significant factor that plays an important role in the seropositivity of dogs to *Brucella* infection. Our finding showed that adult dogs (>3years) were more than six times more likely to be seropositive to antibodies to *Brucella sp* than the younger ones (OR=6.3; 95% CI: 3.3 - 11.6). Previous reports similarly indicated that *Brucella* infection in dogs is age-dependent [2, 6, 28]. This could be as a result of longer exposure period with associated higher risks that adult dogs would have been subjected to, an assertion previously corroborated by Kebede et al. (2008) [36].

The breed specific prevalence showed that the mongrel breed is about 30 times more likely to be infected than the Alsatians. This finding may be as a result of inadequate care and attention that dog owners generally give to mongrels (being a local breed and therefore of less commercial value). This attitude may therefore be responsible for the higher exposure to *Brucella* infection. It is noteworthy however, that all canine breeds are equally susceptible to brucellosis [35]. Our finding is similar to that recorded by Cadmus et al. (2011) [2] but in contrast with that recorded in companion dogs in Ahaz, Iran [6], that did not associate the breed of dogs with seropositivity to antibodies to *Brucella sp*. Based on sex of dogs screened, the females were found to be more seropositive than males, though not statistically significant. This observation is similar to the findings by Cadmus et al. (2011) [2] but contrary to the report of Adesiyun et al. (1986) [37]. This may be because a champion stud is more attractive to breeders. Being a source of income to the owner, such stud is usually mated with many females and therefore putting the females at risk of getting infected [2]. Thus, as neither the stud nor bitch is tested, the infected champion stud transmits the disease to many bitches until it becomes apparent that it has become infertile. Similarly, in uncontrolled mating among stray dogs; there is always the alpha male which mates all the female dogs on heat. Hence, the alpha male could eventually become infected with brucellosis and then transmits same to the females.

Furthermore, results of this study showed that mated animals had lower seroprevalence than the unmated ones, which is in contrast with logical expectation; since brucellosis, can be transmitted during copulation with infected dog. However, since some of the dogs screened may not have been totally confined, it is possible that unwanted mating had taken place without the knowledge of the owners. This observation could therefore have accounted for higher seroprevalence recorded among the "unmated" dogs. Again, confined dogs were found to have higher seroprevalence than the stray dogs, an occurrence which could be attributed to the practice of feeding dogs with foetuses or raw meat. More so, confined dogs may also have higher exposure risk, if at least one of them in the kennel was infected. Despite our findings, this study had its limitations. First, the main screening tests used were the RBT and RSA, while the cELISA and SAT were only used to corroborate results from positive samples. Furthermore, the authors did not carry out bacteriological isolation of *Brucella spp*. This would have provided better insights into the epidemiology of the disease among dogs screened.

## Conclusion

This study recorded seroprevalence of 10.96% and 12.72% using RSA and RBT respectively, thus reiterating the fact that brucellosis is prevalent among dogs screened in south-western Nigeria. We also found that location, age and history of infertility are significant factors for infection with *Brucella sp* among dogs. Our findings therefore call for the need to step up routine screening of dogs and public health enlightenment campaigns among dog owners in order to limit the associated hazards on both humanss and animals. Finally, control of brucellosis in dogs, will go a long way to prevent zoonotic transmission of the disease, and further avert economic losses associated with adverse reproductive performance in dogs.
